# Unveiling caspase-2 regulation by non-coding RNAs

**DOI:** 10.1038/s41419-022-05270-1

**Published:** 2022-09-28

**Authors:** Yun Zhao, Shanel Dhani, Boris Zhivotovsky

**Affiliations:** 1 Institute of Environmental Medicine, Karolinska Institutet, Box 210, 17177 Stockholm, Sweden; 2 Faculty of Medicine, MV Lomonosov Moscow State University, 119991 Moscow, Russia

**Keywords:** Cell death, RNA

## Abstract

Non-coding RNAs (ncRNAs) are a group of RNA molecules, such as small nucleolar RNAs, circular RNAs (circRNAs), microRNAs (miRNAs) and long-noncoding RNAs (ncRNAs), that do not encode proteins. Although their biofunctions are not well-understood, many regulatory ncRNAs appear to be highly involved in regulating the transcription and translation of several genes that have essential biological roles including cell differentiation, cell death, metabolism, tumorigenesis and so on. A growing number of studies have revealed the associations between dysregulated ncRNAs and caspases involved in cell death in numerous human diseases. As one of the initiator and executor caspases, caspase-2 is the most evolutionally conserved caspase in mammals, exerting both apoptotic and non-apoptotic functions. A great deal of studies has shown the involvement of caspase-2 as a tumor suppressor in multiple oncogene-driven cancers, and yet a comprehensive understanding of its biological roles remains largely unknown. In this review, we highlight a compilation of studies focused on the interaction between caspase-2 and miRNAs/lncRNAs in the context of different diseases in order to deepen our knowledge on the regulatory biofunctions of caspase-2 and, furthermore, provide more insight into understanding the role that ncRNAs/caspase-2 axis plays in the development of human diseases.

## Facts

- ncRNAs are a group of RNA molecules which do not encode proteins.
- The regulatory ncRNAs such as miRNAs and lncRNAs are involved in gene regulation and in the development of various diseases.
- Caspase-2 is the most evolutionarily conserved caspase in mammals with its biofunctions being poorly understood.
- Multiple ncRNAs are demonstrated to play an important role in tumorigenesis and other diseases development via caspase-2 regulation.

## Open questions

- Many studies have proposed a functional role for ncRNAs in inducing apoptosis by the modulation of caspase-2 levels. However, considering the diversity of ncRNA targets it is unclear whether these ncRNAs directly regulate the apoptotic functions of caspase-2 or induce apoptosis by a multitude of targets.
- Despite several ncRNAs shown to regulate caspase-2 levels, the mechanism of its regulation and whether these ncRNAs act as a master regulator for caspase-2 remains ambiguous.
- In addition, caspase-2 is implicated in apoptosis and other biological functions, therefore does the regulation by ncRNAs simultaneously affect more than one pathway?

## Introduction

For centuries, messenger RNAs (mRNAs) (as templates of protein synthesis) have become the major research focus for their role in gene regulation and other important biological events, while non-coding RNAs (ncRNAs) (RNA that does not encode proteins) were overlooked because they were considered as “junk” DNA with less biological meaning [1, 2]. However, since the late 1950s, several species of RNA have progressively surfaced, which uncovered a non-coding world. ncRNAs are divided into two functional categories, namely housekeeping and regulatory [3]. The last of them, such as microRNAs (miRNAs), circularRNAs (circRNAs), and long non-coding RNAs (lncRNAs), are significantly involved in gene transcription and translation and form an important component in the regulation of cell differentiation, ontogenesis, inflammation, and angiogenesis [4].

miRNAs are single-stranded small RNA molecules of ~18–25 nucleotides in length that bind to target mRNA and negatively regulate the gene transcript [1]. Interestingly, many lncRNAs (>200 nucleotides in length) resemble mRNAs characteristics in both being 5′capped, spliced, and polyadenylated; but differ in a shorter length, fewer longer exons, and lower expression levels [5]. In addition, their structure is unstable and, therefore, they have a short half-life (<2 h) compared to miRNAs (48 h) [6]. miRNAs are involved in nearly all cellular processes and are essential for development, differentiation and homeostasis [7]. Thus, dysregulation of miRNA functions is associated with several diseases, predominantly with cancer [8]. As such, miRNAs can act as both oncogenes (onco-mirs) and tumor suppressors [9]. CircRNAs have a closed circular structure and lack 3′ and 5′ ends [10]. They have multifunctional roles including regulation of gene expression, protein-protein interactions or are translated into proteins, and have been associated with disease promoting and protective effects [11, 12]. While there is no evidence on the relationship between circRNAs and caspase-2, a few studies have shown alterations to caspase-1, -3 and -9 by circRNAs in cancer and cardiac dysfunction albeit as a downstream effect [13–15].

Likewise, due to their ability to interact with DNA, RNA, and proteins, lncRNAs can regulate diverse cellular processes such as chromatin modification, transcription, post-transcriptional modifications, scaffolding, post-transcriptional mRNA regulation and miRNA regulation [16]. Accordingly, lncRNAs can be found in equally diverse subcellular locations such as in the nucleus, subnuclear domains, and the cytoplasm [17]. Despite their diversity, majority of lncRNAs are unknown, and many lncRNAs are considered to have minor functions. Nonetheless, the mechanistic and functional roles of a handful of classic lncRNAs are well-understood [18]. The lncRNA X inactive specific transcript is a major effector in X chromosome inactivation, HOX transcript antisense RNA (HOTAIR) in positional identity and telomerase RNA component in telomere elongation [19]. Moreover, lncRNAs were found to be directly linked to the transformation of healthy cells into tumor cells [20]. For example, overexpression of the lncRNA HOTAIR is associated with breast cancer metastasis [21].

Caspases are well-defined cysteine proteases primarily involved in inflammation and cellular apoptosis [22]. Amongst all caspases, caspase-2, functionally grouped as an initiator caspase, is the most evolutionally conserved and has been described to fulfill both apoptotic and non-apoptotic functions, including tumor suppression, cell cycle regulation, and DNA repair [23]. Despite its involvement in several cellular processes and identification as a tumor-suppressor in multiple oncogene-driven cancers, a clear functional role of caspase-2 remains unknown. Here, we focus on the interaction between caspase-2 and miRNAs/lncRNAs and discuss whether this could further impact our understanding the regulatory roles of caspase-2 or vice-versa.

## General biofunctions of noncoding RNAs

As mentioned above, miRNAs as well as lncRNAs have been recognized to fulfill a wide range of functions in eukaryotic organisms and, are implicated as fundamental regulators of gene expression in various processes in almost all species [24, 25].

### miRNAs: a key class of small ncRNAs

#### Biogenesis of miRNAs

**Fig. 1:**
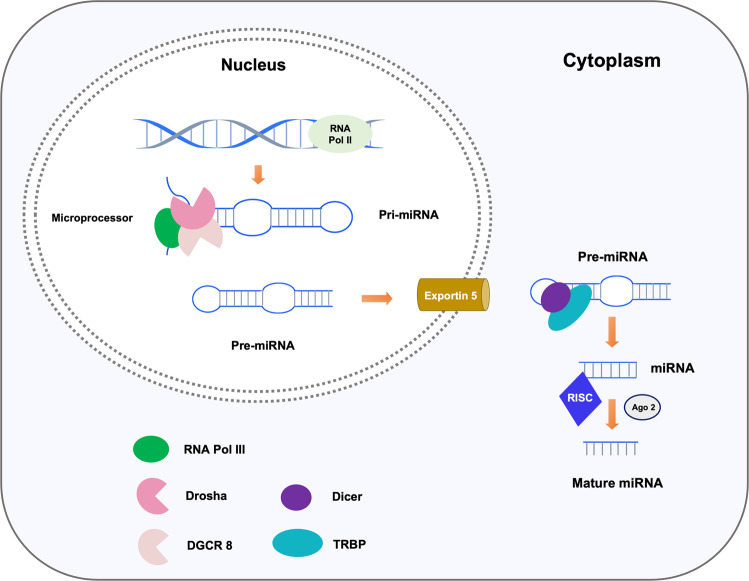
miRNA biosynthesis. Pri-miRNAs are transcribed by RNA Pol II in the nucleus and are subsequently cleaved by the “Microprocessor”, consisting of Drosha and DGCR8, to produce an ~70 nucleotides hairpin like structure, i.e., pre-miRNA. Once formed, pre-miRNAs in the nucleus are translocated by exportin 5 to the cytoplasm, where mature duplex miRNAs are generated through the process of pre-miRNA cleavage by Dicer-TRBP complex. Subsequently, the active miRNA strand (~22 nucleotides) is loaded into RISC together with Ago 2 to exert functions via targeting the downstream mRNAs, whereas the other strand is degraded.

miRNAs are a group of endogenous RNAs which comprise of a prominent class of small ncRNAs in most somatic tissues and play a key role in post-transcriptional gene regulation [26–28]. The biogenesis of miRNAs starts from the generation of stem-loop containing primary miRNAs (pri-miRNAs), which are transcribed by RNA polymerase II (Pol II) in the nucleus [26, 29]. The pri-miRNAs are subsequently cleaved by a so-called “Microprocessor”, a multiprotein complex comprising of Drosha, an RNAse III enzyme, and DGCR8, a double-stranded RNA binding domain (dsRBD) protein, to produce a hairpin like structure called the precursor miRNA (pre-miRNA) with ~70 nucleotides [28, 30–32]. Next, the pre-miRNAs are translocated by exportin 5 from the nucleus to the cytoplasm, where the pre-miRNAs are cleaved by another RNAse III enzyme Dicer together with transactivation-responsive RNA-binding protein (TRBP), a dsRBD protein, to become a mature duplex miRNA [26, 29]. Once the mature double-stranded miRNA is generated, the active miRNA strand (~22 nucleotides), which has relatively lower stability of base-pairing at its 5′ end, is loaded onto the RNA-induced silencing complex (RISC), a ribonucleoprotein complex with TRBP and Argonaute 2 (Ago2) as well as Dicer as the core proteins, while the other strand, the passenger or star (*) strand, is typically degraded [26, 28, 33] (Fig. 1).

#### Biofunctions of miRNAs in regulating gene expression

**Fig. 2:**
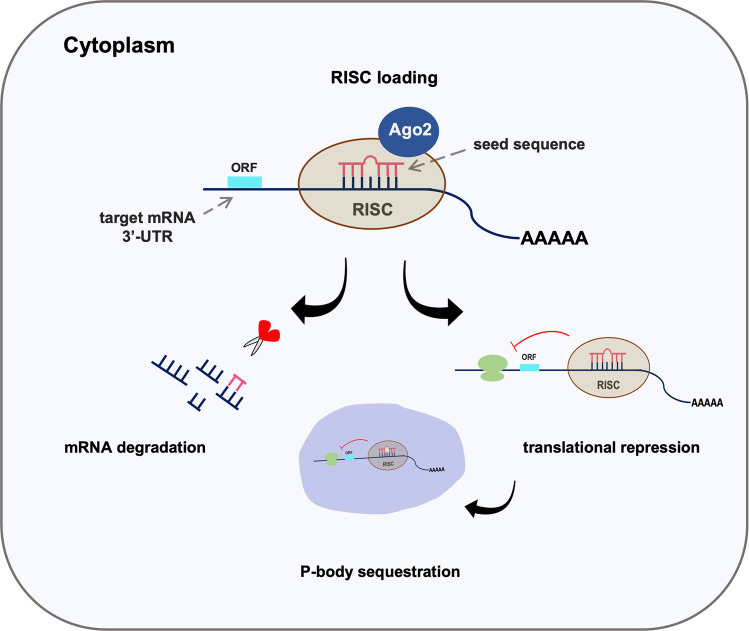
Regulation of gene expression by miRNAs. The “seed sequence” of the guide miRNA strand incorporates into RISC forming miRISC, where the miRNA strand binds to the 3′-UTR of the target mRNA either by perfect or imperfect base-pairing strategies, subsequently resulting in mRNA degradation or translational repression of the corresponding genes.

Over 2600 mature miRNAs have been reported in humans and play crucial roles in multiple biological processes such as cell proliferation, differentiation, signal transduction, cell death, organ development, metabolism, tumorigenesis and so on [34–38]. The main function of miRNAs is to regulate gene expression post-transcriptionally through either canonical or non-canonical mechanisms. In cases of the former mechanism, the guide miRNA strand in the miRNA-induced silencing complex (miRISC), specifically the first 2–7 nucleotides from the 5′ end or so-called “seed sequence”, binds to the 3′-untranslated region (3′-UTR) of the target mRNAs, with a few exceptions, by perfect or near-perfect base-pair interaction inducing the degradation of the selected mRNA [26, 29, 39–41]. It is worth noting that Ago 2 is necessary for the miRISC complex to cleave the mRNA due to its nature as the sole endonucleolytic enzyme [26]. In fact, the canonical splicing mechanism is generally reported in plants with very few cases found in vertebrates [40, 42, 43]. In metazoans, the non-canonical mechanism, which is the imperfect base-pairing of miRNAs with the target mRNA, is the most frequent mechanism by which the miRNAs repress the translation of proteins rather than directly cleaving the mRNAs [41]. The repressed mRNAs are then stored in the processing bodies located in the cytoplasm where the components of the protein translational machinery are excluded, functioning as the main mechanism for suppressing protein translation [44] (Fig. 2). The imperfect-complementary pairs allow a single miRNA to target multiple mRNAs; likewise, one mRNA can be targeted by several miRNAs. Thus, different miRNAs and mRNAs can mutually interact with each other in a much more complex network, making refinement of a defined regulatory role difficult to establish [32, 45].

### Biofunctions of LncRNAs

The diverse activities of lncRNAs affect different aspects of physiology from cell differentiation, growth and responses to various stresses/stimuli, to key roles in the nervous, muscular, cardiovascular, adipose, hematopoietic and immune systems and their associated pathologies [46]. For example, BACE1-AS, antisense of the gene encoding β-site amyloid precursor protein cleaving enzyme 1 (BACE1), promotes BACE1 mRNA stability leading to an increase in the levels of neurotoxic amyloid plaques in the brain of individuals with Alzheimer disease. BACE1-AS can also be detected in the plasma of these individuals, thus serves as a potential disease biomarker [47]. Moreover, lncRNAs have been implicated in every hallmark of cancer cells, from the intrinsic capacity of proliferation and survival, to increased metabolism, and in the tumor microenvironment [48, 49]. For example, lncRNAs are transcriptionally regulated by both oncogenic and/or tumor-suppressive transcription factors such as p53, MYC, estrogen receptor, and signaling cascades such as the Notch pathway [50]. In addition, some lncRNAs are activated by p53 following DNA damage [51]. Although only a small fraction of the thousands of lncRNAs expressed may function at some level in cancer cells, these remain largely understudied, as well as lncRNA responses to therapy [50].

## Regulation of caspase-2 by ncRNAs in different diseases

**Fig. 3:**
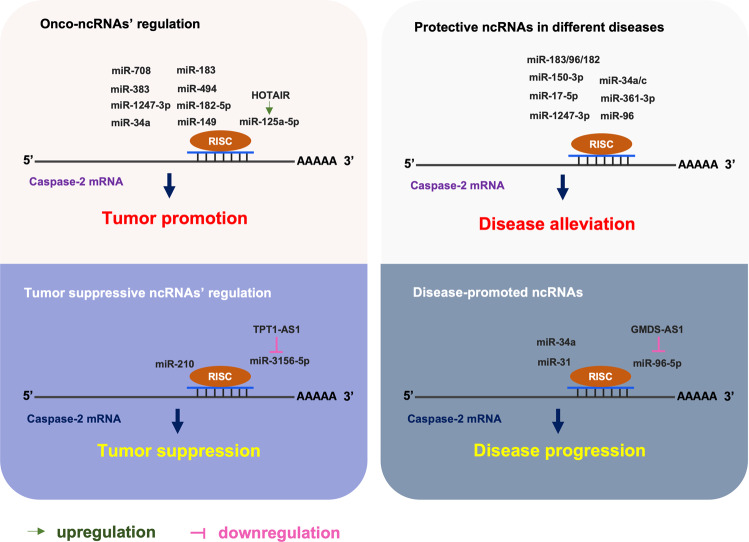
Caspase-2 regulation by ncRNAs in different diseases. For details, see the text.

As the most evolutionarily conserved caspase in mammals, caspase-2 is poorly understood in terms of its functions being controversial in most cases of cell death and its contribution to the development of human diseases [52–54]. On the other hand, the role of ncRNAs have been widely indicated in the pathology of various diseases, particularly cancer [55]. In recent years, an increasing attention has been paid to investigating the role that ncRNAs play in the context of disease processes via the regulation of caspase-2 (Fig. 3).

### Cancer

#### Caspase-2 regulation by miRNAs in cancer

For the first time, Calin et al., (2021) revealed the correlation between miRNAs and cancer and, showed that frequent deletions and the down-regulation of genes encoding miR-15 and miR-16 on chromosome 13 were often found in chronic lymphocytic leukemia patients [56]. The decreased levels of mature miRNAs are generally found in tumors through multiple mechanisms including genomic loss, epigenetic silencing, deregulated biogenesis or transcriptional repression [57, 58]. Genomic expression profiling show that miRNAs exert multifunctional roles in human neoplasms either by targeting the mRNAs of oncogenes, as well as some onco-miRs as tumor suppressors or by having oncogenic roles as onco-miRs in cancer progression [59]. Similar to the data from cancer microarray experiments from the NCBI Gene Expression Omnibus repository, Ren et al. showed a dramatic under-expression of caspase-2 in multiple types of human tumors compared with normal tissues, suggesting the participation of caspase-2 in suppressing tumors [60]. In addition, experiments using caspase-2 null mice showed that this enzyme might act as tumor suppressor via its ability to eliminate cells with chromosomal perturbations or after mitotic insults [52]. Moreover, caspase-2 might help to maintain genomic stability, decreasing the level of oxidative stress and DNA damage [61]. Given the complexity of caspase-2 and the vital regulation of miRNAs in tumor progression, an increasing number of studies have been focusing on these two intriguing regulators in cancer development (Table 1).

**Table 1:** The regulation of caspase-2 by ncRNAs in cancer.

Type	ncRNAs	Cancer	Capase-2 regulation	Effect on tumor	References
miRNAs	miR-708	Bladder carcinoma	Inhibition (↓)	Promotion	[60]
	miR-383	Epithelial ovarian cancer	Inhibition (↓)	Promotion	[61]
		Colon cancer			[62]
	miR-210	Colorectal cancer	Activation (↑)	Suppression	[63]
	miR-1247-3p	Gastrointestinal cancer	Inhibition (↓)	Promotion	[64]
	miR-34a	Liver cancer	Inhibition (↓)	Promotion	[65]
	miR-183	Non-small cell lung cancer	Inhibition (↓)	Promotion	[66]
	miR-494				[67]
	miR-182-5p				[68]
	miR-149	Glioma	Inhibition (↓)	Promotion	[69]
		Acute myeloid leukemia			[70]
lncRNAs/miRNAs	HOTAIR/miR-125a-5p	Colon cancer	Inhibition (↓)	Promotion	[88]
	TPT1-AS1/miR-3156-5p	Breast cancer	Activation (↑)	Suppression	[89]

Despite the low-expression of miRNAs being a common occurrence in cancer, previous studies have shown a significant elevation of miR-708 level in human bladder carcinoma tissue, therefore, suggesting its role in the tumorigenesis of bladder carcinoma [62, 63]. Moreover, bioinformatic analysis and other biochemical approaches, such as luciferase assay and western blotting, revealed caspase-2 as a direct target of miR-708 for bearing the corresponding putative binding site and that the resulting overexpression of caspase-2 was able to offset the anti-apoptotic function of miR-708 in bladder cancer cells [63]. Similarly, miR-383 was demonstrated to be highly expressed in human epithelial ovarian cancer cells [64]. Inhibition of caspase-2, a downstream target of miR-383, was shown to restore the tumor suppressive effect induced by miR-383 down-regulation [64]. In addition, the silencing of miR-383 promoted apoptosis by elevating caspase-2 expression, indicating that caspase-2 might be involved in the cell death induced by miR-383, at least in colon cancer cells [65]. On the contrary, cleavage of caspase-2 and up-regulated expression of Bim, a pro-apoptotic Bcl-2 family protein, was induced by the overexpression of miR-210 in colorectal cancer, resulting in apoptotic cell death [66]. In a recent study of gastrointestinal cancer, miR-1247-3p was shown to directly target caspase-2 by decreasing its levels and the exposure to *Hippophae rhamnoides* L. polyphenols (HPs60) decreased miR-1247-3p levels and correspondingly promoted caspase-2 expression in HPs60-induced apoptotic colon cancer cells [67]. In an in vivo study using hepatocytes of *APC*^KO^ mice model to represent β-catenin activation in liver tumor, administration of an miR-34a inhibitor, a regulatory target of β-catenin, increased caspase-2 activity and showed anti-proliferative effects in β-catenin-mutated liver tumors [68]. Importantly, caspase-2 activity was up-regulated together with β-catenin suggesting that caspase-2 is regulated by miR-34a rather than β-catenin. Moreover, multiple miRNAs including miR-183 [69], miR-494 [70] and miR-182-5p [71] have been shown to function as tumor promoters in non-small cell lung cancer (NSCLC) by inhibiting caspase-2-induced apoptosis. In glioma cells, miR-149 was demonstrated to directly target caspase-2 via the inactivation of p53 and p21 pathways [72]. In addition, the suppression of caspase-2 by miR-149 was also demonstrated in acute myeloid leukemia cells [73].

Bypassing apoptosis is deemed as one of the major hallmarks of cancer progression, in which miRNAs can play a role through regulating the apoptotic process [74]. miRNAs are able to regulate gene expression in both intrinsic and extrinsic apoptotic pathways either directly by influencing the corresponding mRNAs or by affecting other miRNAs to indirectly modulate target genes [75, 76]. For instance, miR-21 was revealed to act as an anti-apoptotic miRNA which directly inhibits FasL to protect cancer cells from undergoing chemotherapy-induced receptor-mediated apoptosis [77], while miR-130a was able to reduce TRAIL resistance in NSCLC cells and activate apoptosis through c-Jun-mediated down-regulation of miR-221 and miR-222 [78].

Caspase-2 is known as an initiator of apoptosis in response to several stimuli, yet the systematic understanding of its physiological role has remained deficient. As aforesaid, in the context of cancer progression, the essence of caspase-2 as a tumor suppressor highlights it as a vital target of miRNAs in the modulation of cancer. So far, research focused on the interaction between miRNAs and caspase-2 in carcinomas is still limited and preliminary. In the above-mentioned studies, most of the miRNAs function as an inhibitor of caspase-2 to exert its anti-apoptotic effects in different cancer types. Likewise, caspase-2 can also be activated by certain miRNAs, such as miR-210 [66]. This miRNA is acknowledged as one of the primary hypoxia-induced miRNAs, and the induction of miR-210 predominantly characterizes cellular response to hypoxic stress [79]. Overexpression of miR-210 is widely found in most solid tumors and acts as an anti-apoptotic regulator in a variety of cell types [79]. However, accumulating evidence revealed that miR-210 may also function as a pro-apoptotic player in different cell contexts [80, 81]. In particular, a recent study demonstrated that miR-210 regulated apoptotic cell death in an opposite manner in cardiomyocytes by alleviating hypoxia-driven intrinsic apoptosis, while significantly promoting the reoxygenation-induced caspase-8-mediated extrinsic apoptotic pathway [82]. Considering that caspase-2 is involved in both intrinsic and extrinsic apoptotic pathways [83] and can be activated by miR-210 overexpression in colorectal cancer, indicates that a more complex role of the miR-210/caspase-2 axis exists in disease development. In addition, it is interesting to further delve into the interaction between caspase-2 and pro-apoptotic miRNAs in the extrinsic apoptotic pathway to perhaps better understand the apoptotic roles of caspase-2.

#### Caspase-2 regulation by lncRNAs in cancer

Recent evidence suggests that lncRNAs form an important component in tumor biology due to their interaction with DNA, RNA and proteins [5]. However, only a small number of lncRNAs have been elucidated for their role in cancer. For example, the lncRNA LINC-PINT inhibits tumor cell invasion through regulating the availability of free PRC2 at the proximity of co-regulated genomic loci [81]. The lncRNA MetaLnc9 facilitates lung cancer metastasis by regulating the AKT/mTOR pathway [82]. The lncRNA FAL1, which is overexpressed in epithelial tumors, is associated with the epigenetic repressor BMI1 and regulates the transcription of several genes, promoting tumor growth [83]. MALAT1 is involved in renal cell carcinoma progression by interacting with EZH2 [84]. The lncRNA EGFR-AS1 mediates epidermal growth factor receptor addiction and modulates the drug resistance of squamous cell carcinoma [85]. The lncRNA PCAT6, involved in the progression of prostate and lung cancer, acts as a key activator of caspase recruitment domain (ARC) by forming a complex with EZH2, inhibiting cell apoptosis and contributing to colon cancer progression [86]. The lncRNA BC200 is elevated in several tumor types and was found to be significantly reduced exclusively in actively proliferating cells by cell cycle arrest, serum deprivation or chemical inhibition [87]. Despite the aberrant expression of lncRNAs in cancer, the increasing discovery rate of new lncRNAs and their diversity of targets presents a huge challenge in understanding its significance and necessitates extensive research before it can be clinically translated.

As aforementioned, caspase-2 is well-established for its role in apoptosis and as a tumor suppressor. However, the regulation of its various functions is poorly understood. In some cases, the modulation of miRNAs on caspase-2-initiated apoptotic cell death may be regulated by certain lncRNAs (Table 1). Knockdown of *HOTAIR*, a lncRNA frequently overexpressed in various carcinomas and potential anti-cancer target, was shown to decrease miR-125a-5p and increase caspase-2 cleavage resulting in apoptotic death [88]. The authors also identified p53 as a target of miR-125a-5p and was upregulated upon HOTAIR knockdown [88]. However, apoptosis was inhibited by caspase-2 silencing in HOTAIR knockdown models revealing its significance in inducing apoptosis by targeting HOTAIR in cancer [88]. Recently, the overexpression of lncRNA TPT1-AS1 was shown to repress cell proliferation and sensitize breast cancer cells to paclitaxel by elevating caspase-2 levels via the sponging of miR-3156-5p [89]. This effect was decreased by the silencing of caspase-2 indicating the significance of caspase-2 in inducing breast cancer sensitivity to paclitaxel [90]. It is worth noting that the levels and function of TPT1-AS1 are different in various cancer types. For example, TPT1-AS1 promotes the proliferation of colorectal, ovarian and cervical cancers, therefore, targeting of TPT1-AS1 may be cancer type specific [89, 91, 92]. Interestingly, TPT1-AS1 was found to be predominant in the cytoplasm [90]. Given that caspase-2 and lncRNAs are present in different cellular compartments it will be interesting to explore their interaction in the different compartments in healthy cells comparative to disease-type cells in hope that this may provide insight into the regulatory switch or the role that caspase-2 plays in cancer development/elimination [93–95]. Collectively, the lack of studies and the differential correlation between levels of lncRNA and caspase-2 among cancer cell types make it difficult to conclude on the precise mechanisms of caspase-2 regulation by lncRNAs. It may be of value to further understand the relationship between lncRNA and miRNA seeing as lncRNA indirectly regulate caspase-2 via miRNA, again cellular localization of this may dictate the functional role of caspase-2.

### Other diseases

#### Caspase-2 regulation by miRNAs in other diseases

- **Ophthalmic degeneration**
  In addition to cancer, ongoing studies are also committing to exploring the role of miRNAs/caspase-2 interaction in other diseases (Table 2). Previously, a sponge transgenic mouse model was constructed by disrupting activities of the miR-183/96/182 cluster in the retina to investigate how these miRNAs impact on retinal maintenance and light adaptation [96]. Severe retinal degeneration was observed in the transgenic mice after intense light exposure and a number of genes including *CASP2* were identified as potential targets of this cluster (Zhu et al., 2011). In this study, caspase-2 was upregulated upon the simultaneous disruption of the miR-183/96/182 cluster in the transgenic mouse model. Moreover, the inhibition of caspase-2 with Z-VDVAD-FMK partially rescued the retinal damage induced by the acute light exposure, indicating that the miR-183/96/182 cluster prevents light-induced retinal degeneration by mediating caspase-2 expression [96]. In another ophthalmic study, miR-96 overexpression-induced apoptosis was shown to be linked to caspase-2 activation in RGC-5 cells, a rat retinal ganglion cell line where caspase-2 was directly targeted by miR-96, suggesting that miR-96 may functionally regulate cell proliferation through caspase-2 in RGC-5 cells [97].

**Table 2:** The regulation of caspase-2 by ncRNAs in other diseases.

Type	Disease	ncRNAs	Capase-2 regulation	Effect on diseases	References
miRNAs	Ophthalmic degeneration	miR-183/96/182 cluster	Inhibition (↓)	Protection	[96]
		miR-96	Activation (↑)	N/A	[97]
	Neuronal damage/diseases	miR-1247-3p	Inhibition (↓)	Protection	[99]
		miR-17-5p	Inhibition (↓)		[100]
		miR-150-3p	Inhibition (↓)		[101]
		miR-34a/c	Inhibition (↓)		[104]
	Liver diseases	miR-31	Inhibition (↓)	Promotion	[105]
		miR-34a	Inhibition (↓)		[106]
		miR-96	Inhibition (↓)	Protection	[108]
	Myocardial damage	miR-1247-3p	Inhibition (↓)	Protection	[126]
		miR-361-3p	Inhibition (↓)		[127]
lncRNAs/miRNAs	Sepsis	GMDS-AS1/miR-96-5p	Activation (↑)	Promotion	[128]
  In both ophthalmic studies, miR-96 was revealed to be a key regulatory element of caspase-2 in vivo and in vitro albeit in opposed manners. As for the miR-183/96/182 cluster, a sensory organ-specific paralogous cluster of which the three component miRs’ sequences are similar to each other [98], repression of caspase-2 expression largely explained how this specific cluster could protect the retina from light-induced degeneration [96]. In addition, miR-96 and miR-183 but not miR-182 were demonstrated to be primarily involved in retinal protection due to the up-regulation of only the prior two miRs but not miR-182 after strong light exposure even though miR-183 was not predicted to target the mRNA of caspase-2 [96]. In addition, despite the up-regulation of caspase-2 using miR-96-mimics in RGC-5 cells, the authors failed to specify the isoform of caspase-2 (cleaved or total) investigated, as this is important in understanding the function and significance of caspase-2 in apoptosis. Nor was a plausible explanation given for obtaining contradictory results from the other study, which showed decreased levels of both pro- and cleaved caspase-2 in a transgenic mouse model. Considering the variances in the models used in the two studies, more extensive work is required to clarify the regulatory mechanism of miR-96 on caspase-2 in the ophthalmic system.
- **Neuronal damage/diseases**
  The association of miRNAs with caspase-2 activity has also been reported in neuronal damage/diseases. In a cerebral stroke study, down-regulation of miR-1247-3p, one of the regulatory miRNAs targeting caspase-2, resulted in the up-regulation of caspase-2 during ischemia/reperfusion (I/R) injury in stroke, as well as, in N2a cells treated with oxygen-glucose deprivation/reoxygenation (OGD/R), an in vitro model for I/R injury [99]. Moreover, elevation of miR-1247-3p levels was able to diminish apoptosis in OGD/R model by inhibiting caspase-2 expression, suggesting that miR-1247-3p acts as an upstream regulator of caspase-2 and, plays a protective role in brain I/R injury via caspase-2 repression [99]. A recent study reported high levels of caspase-2 in hypoxia-ischemia brain damage (HIBD) mice with a low expression of miR-17-5p, which was found to target and negatively regulate caspase-2 [100]. Overexpression of caspase-2 was able to block the effects of miR-17-5p mimics on brain tissue damage alleviation, as well as the improved memory ability in the HIBD mouse model, indicating the protective effect of miR-17-5p on HIBD by inhibiting caspase-2 [100].
  Recently the neuroprotective role of stem cell-derived exosomes in hypoxic-ischemic brain injury was shown [101]. miR-150-3p was identified as the most abundantly expressed miRNA in exosomes compared to their parent neuronal stem cells and *CASP2* was predicted as a miR-150-3p target. Interestingly, the miR-150-3p mimic fulfilled the neuroprotective effects while miR-150-3p inhibitor exacerbated nerve injury both in vivo and in vitro. It seems that stem cell-derived exosomes are able to facilitate the neuroprotective effects via transfer of miR-150-3p which targets *CASP2*, thus suppressing neuronal apoptosis after brain injury. It will be interesting to investigate whether this mechanism can be used in the future for prevention of cerebral injury.
  Lidocaine has been reported to directly target spinal cord dorsal root ganglion neurons (DRGNs) via spinal and epidural administration, causing neurotoxicity such as neuronal apoptosis, neurite growth repression, etc [102, 103]. Up-regulation of miR-34a/c, but not miR-34b, was able to protect DRGN apoptosis upon lidocaine treatment by inhibiting caspase-2, implying that miR-34a/c may function as an upstream regulator of caspase-2 to alleviate lidocaine induced neurotoxicity [104]. In brief, the functional interaction of the miR-34 family and caspase-2 is apparent in different disease states, either acting as an onco-miR negatively affecting health, or, on the other hand, protecting against neuronal damage. Despite the significance of the interaction in the different states, it is evident that miR-34 plays a regulatory role of caspase-2-induced apoptosis.
- **Liver diseases**
  Mounting evidence has revealed the regulation of caspase-2 by miRNAs in liver diseases. It was reported that *Staphylococcal* enterotoxin B induced acute liver inflammation and injury and could be attenuated by natural indoles through the down-regulation of miR-31 expression and the consequent activation of caspase-2-dependent apoptosis in T cells [105]. As one of the dominant targets of miR-34a, caspase-2 was found to be decreased in liver tissues of ethanol-treated mice along with a marked increase of miR-34a level, indicating the involvement of miR-34a/caspase-2 association during alcoholic liver injury [106]. On the other hand, caspase-2 expression is linked with the apoptotic cell death in non-alcoholic fatty liver disease (NAFLD) and non-alcoholic steatohepatitis (NASH) [107]. A significant decrease of miR-96-5p was observed in a high fat diet induced NASH model, while bone marrow mesenchymal stem cells (BM-MSCs) and their derived exosomes (BM-MSCs-Exo) were able to serve as a therapeutic treatment for NASH via up-regulating miR-96-5p, which subsequently inhibited caspase-2 to prevent liver apoptosis, hyperlipidemia and hepatic steatosis [108].
  miR-96 is one of the liver-specific miRNAs and serves as a potential biomarker for liver injury (mainly via apoptosis, necrosis and necroptosis) [109] and is frequently implicated in different liver conditions such as advanced liver fibrosis [110], hepatocellular carcinoma [111], and aging-related NAFLD [112]. Moreover, up-regulation of the miR182/96/183 cluster has been reported in non-viral hepatocellular carcinoma (NBNC‐HCC) [113], as well as, in chronic liver diseases such as hepatic fibrosis [114]. The regulation of caspase-2 by this cluster, dominantly by miR-183 and miR-96, has been demonstrated in retinal protection [96], which raises several more questions regarding the interaction of the miR-96/caspase-2 axis, e.g., are other components of the cluster also contributing to the miR-96-mediated caspase-2 interaction in the context of multiple liver diseases? If yes, considering that miR-183 is also described as a liver-specific miRNA [109], does miR-183 also function as a primary co-regulator along with miR-96, or does the whole cluster work as an entirety? Could any other liver-specific miRNAs potentially regulate caspase-2 in the progression of different liver disorders?
  miR-34 family comprising of miR-34a, miR-34b and miR-34c was the first miRNA family genes identified to be directly regulated by p53 with miR-34a being the most highly regulated miRNA [115–119]. Despite miR-34 functioning mainly as a tumor suppressor by repressing more than 700 transcripts that are involved in cellular proliferation, survival and plasticity [120], the up-regulation of miR-34 family genes was also reported in different cancers such as osteosarcoma [121] and liver cancer [122, 123] as aforementioned. It is reasonable that the significance of caspase-2 apoptotic roles in the interaction between miR-34 and caspase-2 were determined in disease states governed by the imbalance of cell death and survival/proliferation (cancer and neuronal damage); but perhaps, given the type of the disease, may also have an effect on other caspase-2 functional roles such as metabolism since liver disease is also categorized as a metabolic disorder [124] and caspase-2 has been established for its metabolic roles [125].
- **Myocardial damage**
  In hypoxia/reoxygenation (H/R)-treated H9c2 cells, an in vitro model mimicking myocardial I/R injury using rat cardiomyocytes, miR-1247-3p [126] and miR-361-3p [127] were down-regulated. Overexpression of these two miRNAs was indicated to release H/R-induced injury in H9c2 cells by inhibiting caspase-2-initiated apoptosis, implying a cardioprotective effect of these two miRNAs on I/R-induced myocardial damage [126, 127]. Although the obtained results are interesting, presently it is unclear whether these observations may lead to the development of novel therapeutics for hypoxia-related cardiac diseases.

#### Caspase-2 regulation by lncRNAs in other diseases

Sepsis is a life-threatening inflammatory disease and has a higher mortality rate than breast and lung cancer [128]. While the pathogenesis of sepsis remains poorly understood, aberrant expression of lncRNA has been implicated in the progression of the disease [128] (Table 2). In addition, certain lncRNAs and miRNAs have been shown to have regulatory roles in inflammatory responses and apoptosis to slow down the development of sepsis [128]. The lncRNA GMDS‐AS1, which inhibits cell proliferation and induces apoptosis by targeting the miR‐96‐5p/CYLD axis in lung adenocarcinoma, was also found to inhibit miR‐96‐5p expression in a sepsis model [128]. The authors further identified caspase-2 as a target of miR‐96‐5p and was negatively modulated by miR‐96‐5p [128]. While the regulatory role of GMDS-AS1 and miR-96-5p on caspase-2 expression was shown, the authors did not evaluate the significance of caspase-2 in the resultant inflammatory response and apoptotic effects using, for example, caspase-2 loss of function approaches (chemical or genetic) to truly determine whether the outcome was due to caspase-2 or perhaps another more significant target of miR-96-5p. On the contrary, a study by Shalini et al., [129] showed an anti-inflammatory response in caspase-2 knockout mice exposed to reactive oxygen species. Although the models in each study were different, it further begs the question on the magnitude of caspase-2 as a downstream target in these roles and if these roles differ in altered states such as diseases. It is also possible that caspase-2 may have regulatory roles on GMDS-AS1/miR-96-5p. Therefore, it is important to understand if the GMDS-AS1/miR-96-5p/caspase-2 axis has a feedback loop and how other factors may alter components of the axis and its functions.

## Conclusions

Several interesting questions regarding the relationship between ncRNAs and caspase-2 remain unanswered. Indeed, while the interaction between miRNAs and lncRNAs play an important role in various biological processes and in the development of diseases including cancer, detailed molecular mechanisms on their functions and on their ability to function together are poorly understood. It will also be interesting to determine how the related functions between miRNAs/lncRNAs and their interacting protein targets are related to their presence in the cytoplasm or nucleus. However, this is a huge task as there are many targets by individual miRNAs/lncRNAs which makes it challenging to understand and refine for disease treatment. The studies mentioned above have shown the regulation of caspase-2 by miRNA-96-5p and the miRNA-34 family in various cell types suggesting that the interaction between these miRNAs and caspase-2 is not dependent on cell type or disease. Conversely, cell death seems to be the common factor in all the findings. However, the lack of functional studies raises concerns whether the cell death was dependent on the miRNA/caspase-2 interaction. In addition, most of the reported data are isolated research findings by single groups and thus lacks independent validations. Although the role for caspase-2 in apoptosis is established, it is important to consider other caspases or proteins that may fulfill a more dominant or simultaneous function in the outcome observed. Perhaps overlapping roles/mechanisms between miRNA/lncRNA and caspase-2 may provide a better insight into this, such as DNA damage response, genomic stability maintenance, metabolism and cell death. Moreover, many of the studies only examined the regulation of caspase-2 by ncRNAs but, it is also possible that caspase-2 may influence miRNA/lncRNA levels and their function or targets thereof. Nevertheless, these studies reveal ncRNAs as novel regulators of caspase-2 and warrants further investigation into the possibility of the direct, simultaneous or compensatory regulatory mechanisms of caspase-2 and vice-versa.

## Data Availability

The authors have no data to deposit on a repository.
